# Supplementary material for: Cell-intrinsic ceramides determine T cell function during melanoma progression
Source: eLife. 2022 Nov 25;11:e83073. doi: 10.7554/eLife.83073 (PMC9699697; doi:10.7554/eLife.83073)
Supplement: Figure 6—source data 1. [file elife-83073-fig6-data1.zip › Figure 6 - Source data Blots.pptx]

## Slide 1
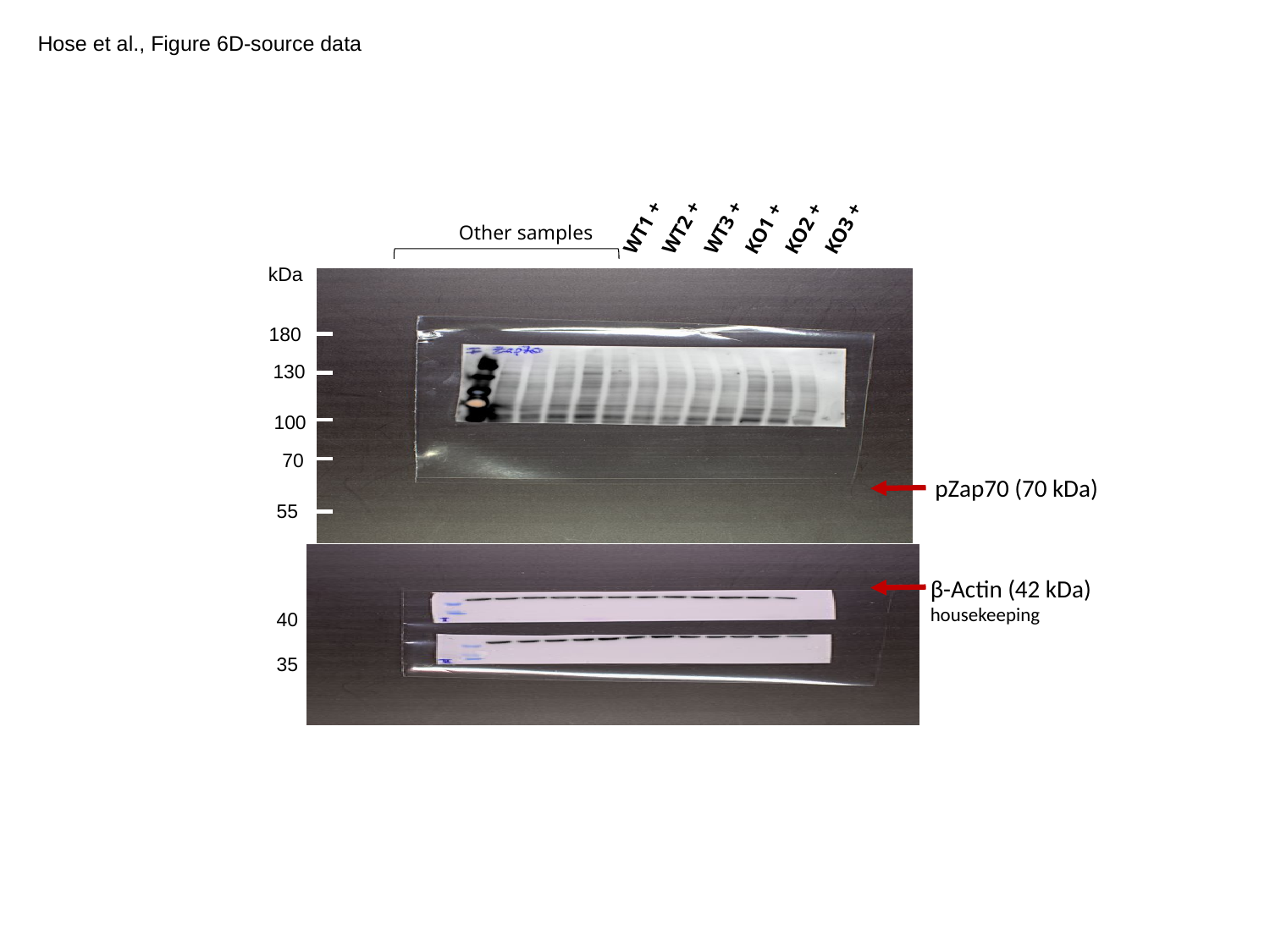

Hose et al., Figure 6D-source data
Other samples
WT1 +
KO1 +
WT2 +
WT3 +
KO2 +
KO3 +
kDa
180
130
100
70
55
40
35
pZap70 (70 kDa)
β-Actin (42 kDa)
housekeeping
