# Supplementary figures and images for: Cell-intrinsic ceramides determine T cell function during melanoma progression
Source: eLife. 2022 Nov 25;11:e83073. doi: 10.7554/eLife.83073 (PMC9699697; doi:10.7554/eLife.83073)

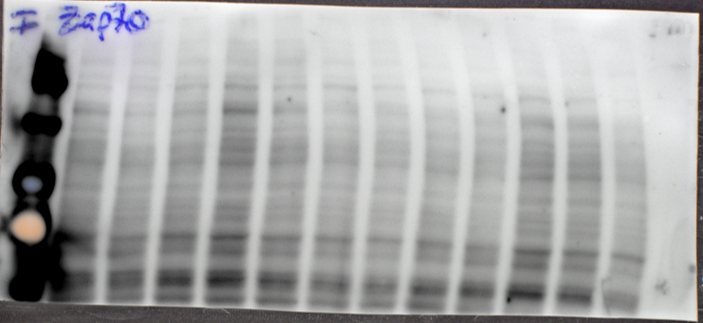

Supplement: Figure 6—source data 1. [file elife-83073-fig6-data1.zip › Blot pZAP70 original.tif]

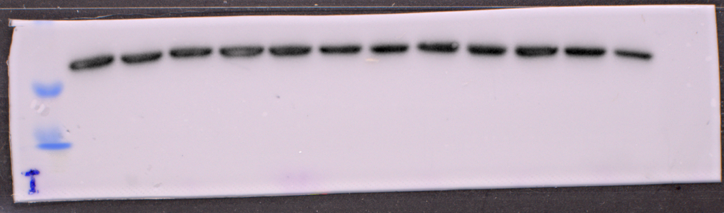

Supplement: Figure 6—source data 1. [file elife-83073-fig6-data1.zip › Blot ß-Actin original.tif]

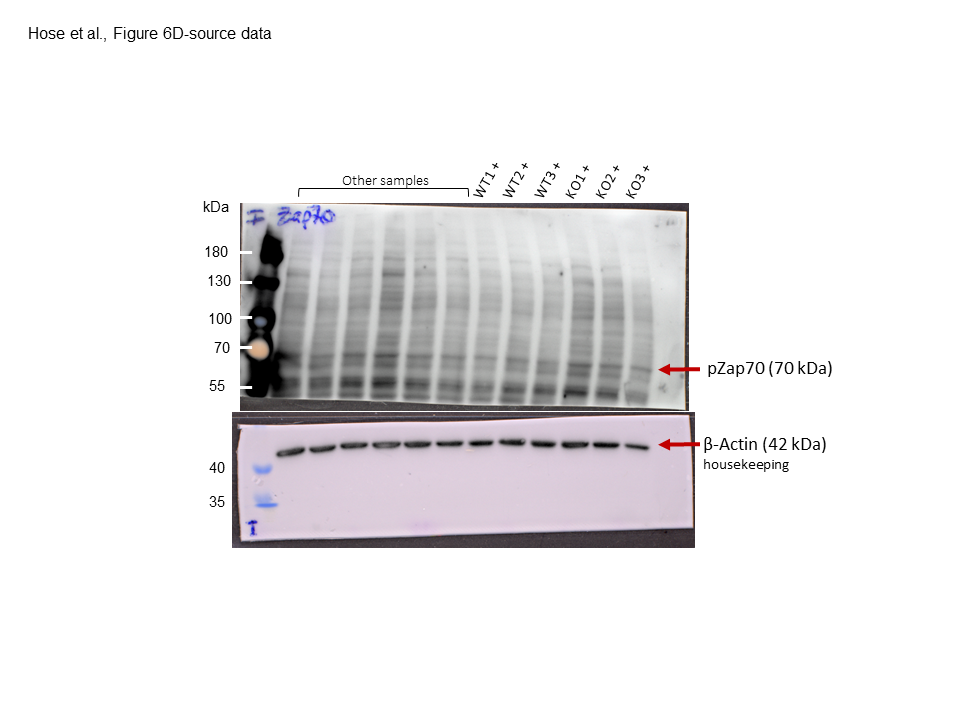

Supplement: Figure 6—source data 1. [file elife-83073-fig6-data1.zip › Figure 6 - Source data_Blot.TIF]
